# Characterization of the complete chloroplast genome of *Corydalis bungeana* Turcz

**DOI:** 10.1080/23802359.2021.1925984

**Published:** 2021-06-14

**Authors:** Qi Wang, Zhixian Lei, Lirong Zhou, Biwei Mai, Naiyun Zhu, Xiaoli Zhao, Wenting Xu

**Affiliations:** a Department of Critical Medicine, Hainan Maternal and Children’s Medical Center, Haikou, P.R. China; b Department of Chinese medicine, Hainan Maternal and Children’s Medical Center, Haikou, P.R. China

**Keywords:** *Corydalis bungeana*, chloroplast genome, Papaveraceae, phylogenetic analysis

## Abstract

*Corydalis bungeana* Turcz. is a perennial herb belonging to the family Papaveraceae. Its chloroplast genome was sequenced and characterized. The cp genome of *C. bungeana* is 167,629 bp long with a GC content of 36.52%. A total of 144 genes were identified in this cp genome, including 79 protein-coding genes, 31 tRNAs and four rRNAs. A phylogenetic tree based on the complete nucleic acid sequence indicated that *C. bungeana* was classified into Corydaleae and had a close relationship with *Lamprocapnos spectabilis*.

*Corydalis bungeana* Turcz., a perennial herb, is classified into the family Papaveraceae (Chase et al. 2016). Previous studies have indicated that special active ingredients from dried whole plants show potential pharmacological activities, including anti-inflammatory, antibacterial and antinociceptive activities (Tian et al. 2019), but limited genomic and genetic resources have impeded the precise identification of *C. bungeana* until now. Herein, we determined the complete chloroplast genome of *C. bungeana*, which will provide informative data for determining the phylogeny of Corydalis and the molecular identification of *C. bungeana*.

Fresh leaves of *C. bungeana* were collected from an individual plant in a greenhouse in Chengxi District (110°19.245′ E, 19°59.757′N), Haikou, China. Fresh leaves disinfected with 75% ethyl alcohol were immediately frozen in liquid nitrogen and stored in the refrigerator at −80 °C for genomic DNA extraction. High molecular weight plant genomic DNA was extracted using a modified CTAB method (Ding et al. 2020) and then stored at the herbarium of Hainan Maternal and Children's Medical Center (http://www.hnwcmc.com/, Qi Wang, wqi1220@163.com) under voucher number 10_1. The pure genomic DNA was sequenced (insert size ∼350 bp) on the Illumina NovaSeq platform according to the standard protocol. Approximately 2.90 Gb raw data were obtained with a paired-end read length of 150 bp, and these raw data were filtered for high-quantity clean reads. Raw reads were deposited in the NCBI Sequence Read Archive database. The cp genome was assembled by using SPAdes v3.13.0 (Bankevich et al. 2012), and this draft cp genome was subsequently corrected based on GapCloser v1.12. The annotation was performed using PGA (Qu et al. 2019) and then submitted to GenBank (accession no. MW538958).

This cp genome of *C. bungeana* was 167,629 bp in length with a GC content of 36.52%, and the base composition was asymmetric (17.83% C, 17.29% G, 3012% A, and 30.91% T). This cp genome of *C. bungeana* was a circular molecule with a small single copy region (SSR, 18,024 bp) and a pair of inverted repeats (IR, 26,411 bp each) separated by two large single copy regions (LSC, 96,783 bp). This cp genome contained 114 genes, 79 of which were protein-coding genes, 31 were tRNA genes and four were rRNA genes. Two genes contained two introns, and nine genes had one intron, whereas 16 genes had two copies in the cp genome of *C. bungeana*.

To determine its phylogenetic position within Rhoeadales, a neighbor-joining (NJ) tree was reconstructed with MEGA X (Kumar et al. 2018) using the complete cp genome sequence of *C. bungeana* and the sequences from 18 additional species from Rhoeadales (Figure 1). The results suggested that *C. bungeana* was separated from other species of *Corydalis* though it was classified into the genus of *Corydalis* yet and the previous study with DNA barcoding also demonstrated that *C. bungeana* was classified into a single clade in phylogenetic tree of *Corydalis* based on ITS and *matK* (Ren et al. 2019). This evolutionary analysis also provided the evidence for Corydaleae had a close relationship with *Lamprocapnos spectabilis* from the Fumarioideae (Papaveraceae) clade (Lidén et al. 1997; Xu and Wang 2021).

**Figure 1.**
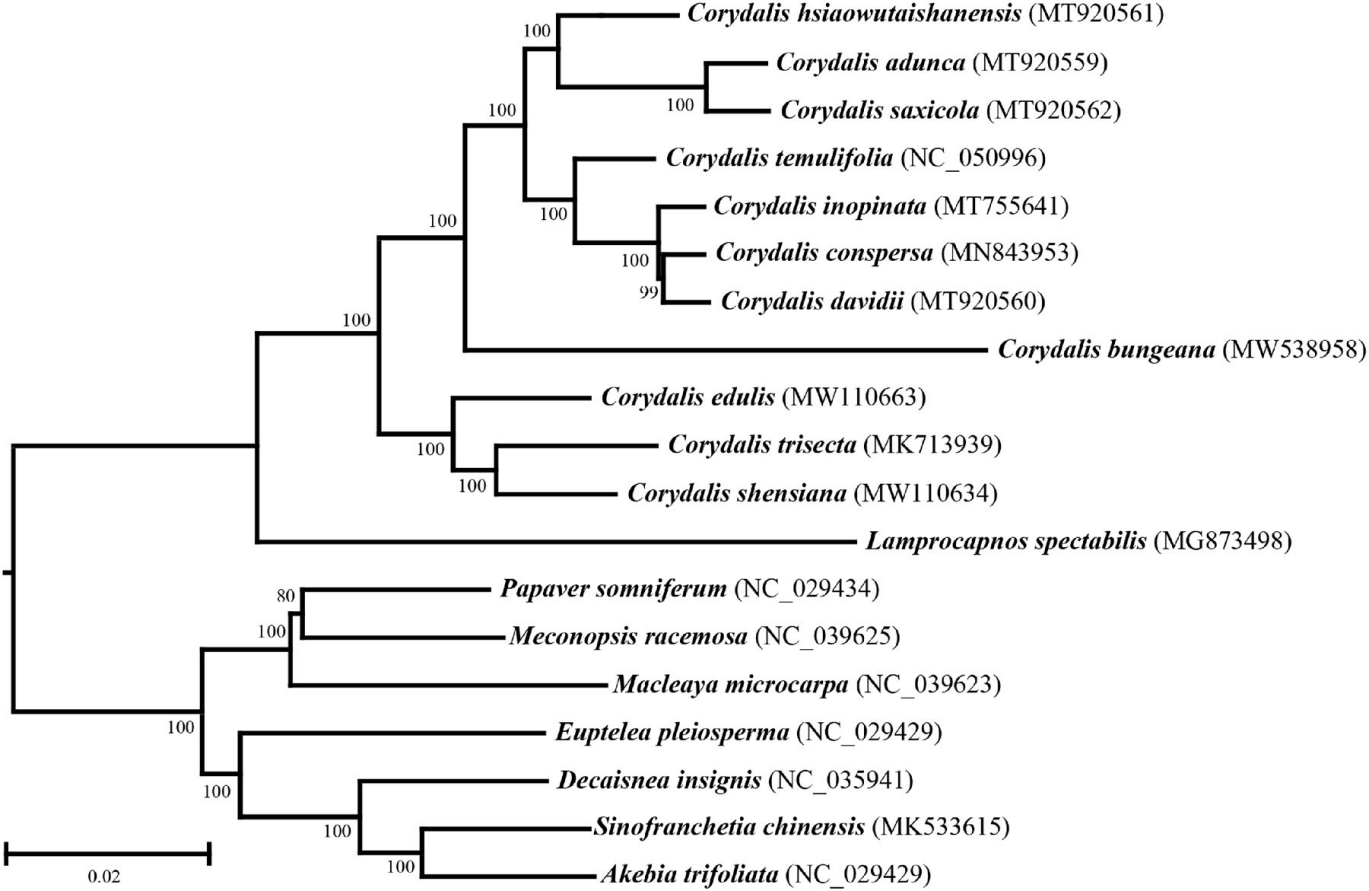
The phylogenetic of 19 species in Rhoeadales based on neighbor-joining (NJ) analysis of the whole cp genome sequence.

## Data Availability

The data that support the findings of this study are openly available in the US National Center for Biotechnology Information (NCBI database) at https://www.ncbi.nlm.nih.gov/, reference number: MW538958. The associated BioProject, BioSample and SRA numbers are PRJNA692558, SAMN17348915, SRR13447699 and SSR13447700.
